# Transcriptomic profiling and IgM and IgT repertoire dynamics in rainbow trout gills following primary and secondary challenge with *Lactococcus petauri*

**DOI:** 10.3389/fimmu.2026.1863531

**Published:** 2026-06-16

**Authors:** Aitor Arrogante, Esther Morel, Beatriz Abós, Samuel Vicente-Gil, Pablo Jiménez-Barrios, Patricia Díaz-Rosales, Pedro Perdiguero, J. Germán Herranz-Jusdado, Carolina Tafalla

**Affiliations:** 1 Fish Immunology and Pathology Group, Biotechnology Department, National Institute for Agricultural and Food Research and Technology (INIA), Spanish Research Council (CSIC), Madrid, Spain; 2 PhD School, Faculty of Sciences, Autonomous University of Madrid, Madrid, Spain; 3 Malaga Oceanographic Centre (COMA), Spanish Institute of Oceanography (IEO), Spanish National Research Council (CSIC), Malaga, Spain; 4 Department of Genetics, Physiology and Microbiology, Faculty of Biological Sciences, Complutense University of Madrid, Madrid, Spain

**Keywords:** bacterial infection, gills, IgM, IGT, secondary responses

## Abstract

Fish mucosal tissues lack the organized lymphoid structures present in mammals. Although this is also true for the gills, with the gill-associated lymphoid tissue (GIALT) being mainly composed of B and T cells scattered throughout the epithelium and the lamellae, in some species such as rainbow trout (*Oncorhynchus mykiss*) or Atlantic salmon (*Salmo salar*), a more organized structure designated as the interbranchial lymphoid tissue (ILT) is also identified. Yet, although many studies have investigated the immune response of the gill to stimulation or pathogen encounter, which have revealed the activation of both innate and adaptive immune elements, how the gills respond to secondary stimulation or whether memory responses are locally orchestrated is still unknown. To provide insights into this matter, in the current study, we have infected rainbow trout by bath with *Lactococcus petauri* and then re-infected or mock-infected the survivor fish as well as mock-infected controls. At day 4 after the last infection, the fish were sacrificed and gills used to perform an RNAseq transcriptomic analysis and an IgM and IgT repertoire analysis. The number of cells secreting total and specific IgM in gills was also established by ELISpot. Our results demonstrate that memory responses are locally organized in the rainbow trout gills, given that re-infected fish did not up-regulate genes related to inflammation (as happens in the primary infection), but preferentially modified genes related to adaptive immunity, mainly to B cell function, experiencing a significant IgM and IgT clonal expansion. Accordingly, re-infected fish produced increased levels of specific IgM both locally and in serum, although no clonal selection was apparent. These findings advance our understanding of mucosal immunological memory in teleost fish and reveal the gills as a site of localized adaptive immune regulation during secondary responses.

## 1 Introduction

Mucosal surfaces represent the first line of defense between the host and the environment and, for this reason, the immune responses organized locally in these tissues constitute a key factor to block the entry of pathogens and prevent their dissemination inside the host. In fish, some of these mucosal surfaces, such as gills, skin or gut are continuously exposed to an environment that has a large antigenic load, including potentially pathogenic microorganisms. Within these mucosal surfaces, innate immune mechanisms provide an initial non-specific line of defense. These innate immune elements include the mucus layer in which antimicrobial peptides, lysozyme or complement proteins act on the potential pathogens. Resident innate immune cells in mucosal surfaces include macrophages, neutrophils or dendritic cells (DCs) which can recognize conserved antigens in pathogens through pattern recognition receptors (PRRs) such as toll-like receptors (TLRs) (1). Through this recognition, a signaling cascade is activated, ultimately triggering a rapid inflammatory response characterized by the early release of pro-inflammatory cytokines such as interleukin 1β (IL-1β) or tumor necrosis factor α (TNF-α) or chemokines such as IL-8 which recruit leukocytes to the site of infection (2). This recognition also involves the activation of antimicrobial pathways in these innate immune cells, such as the production of reactive oxygen species (ROS) or phagocytic processes to eliminate the pathogens at this initial site of replication (3).

While innate immunity ensures immediate protection, adaptive immunity provides antigen-specific recognition, clonal expansion and immunological memory. In this context, fish mucosal surfaces also contain adaptive immune cells (namely B and T cells) that contribute to clearing the pathogens while organizing more specific responses. Within the adaptive immune compartment, T cells are either responsible for helping B cells (CD4^+^ T helper cells) or for recognizing and eliminating infected or tumoral cells (CD8^+^ cytotoxic T cells). B cells, on the other hand, play a central role as producers of immunoglobulins (Igs). These Igs can be expressed on the cell surface acting as a B cell receptor (BCR) or can be secreted, being them usually referred to as antibodies. These antibodies specifically neutralize pathogens. Structurally, Igs are composed of two heavy chains (IgH) and two light chains (IgL). The constant region of the IgH defines the isotype and is shared among antibodies of the same class, whereas the variable region, formed by both regions in both the IgH and IgL chains, confers the antigen specificity. This variable region is obtained by recombination of different V(D)J segments (4). In teleost fish, three different Igs have been identified: IgM, IgD and IgT. The heavy chains of IgM and IgD in teleost are produced through alternative splicing of a shared primary transcript, as occurs in mammals. As a result, both isotypes, display the same variable region within an individual B cell (5). However, IgT originates from a distinct rearrangement process. Because IgT utilizes its own D and J gene segments, its antigen receptor repertoire is generated independently from that of IgM and IgD (6). Therefore, B cells exclusively expressing IgT constitute an independent B cell lineage (7), not one that appears as a consequence of class switch recombination (CSR) as is the case for mammalian IgA.

In systemic lymphoid tissues, the predominant B cell population in teleost consists of mature naïve IgM^+^IgD^+^ cells. Following antigen encounter, these cells typically down-regulate IgD expression and differentiate into IgM^+^IgD^-^ plasmablasts and eventually terminally differentiated plasma cells, resembling the differentiation pathway observed in mammals (8, 9). Both plasmablasts and plasma cells have an increased capacity to secrete antibodies, but differ in their capacity to proliferate (10). Interestingly, recent results from our group have demonstrated that most of the IgM^+^ B cells present in homoeostasis in mucosal surfaces are IgM^+^IgD^-^ B cells that have already initiated a differentiation process, probably due to the normal exposure to antigens in the water or the microbiota (9). Additionally, IgT^+^ cells are a big population of the fish mucosal surfaces. In some of the fish species in which this has been investigated, including rainbow trout (*Oncorhynchus mykiss*), the ratio of IgT^+^ B cells to cells of the IgM/D lineage is higher in mucosal than in systemic tissues (7, 11, 12). For this reason, IgT was initially considered an Ig specialized in mucosal responses or even a functional analog of mammalian IgA, although it seems clear nowadays that this is dependent on the fish species, the tissue, or the antigen that triggers the response (13, 14). Finally, in some species such as rainbow trout, catfish (*Ictalurus punctatus*) or Atlantic salmon (*Salmo salar*), cells exclusively expressing IgD (IgD^+^IgM^-^ B cells) have also been reported (9, 14–17). Interestingly, in salmonids, these cells are frequent in the mucosal tissues, including the gills (9, 14, 16), although their precise role in mucosal immunity is still unknown.

In mammals, adaptive cells within mucosal tissues are usually organized in complex structures that promote the close interaction of B and T cells, being these structures referred to as mucosa-associated lymphoid tissue (MALT). Although this name is also used to designate the lymphoid cells present in the fish mucosae, these cells are often dispersed throughout the tissue in a seemingly disorganized fashion (18). In gills, the lymphoid cells are specifically designated as the gill-associated lymphoid tissue (GIALT) which encompasses B and T cells diffusely distributed within the gill epithelium and lamellae (18). Interestingly, in 2008, Haugarvoll and collaborators identified a more organized structure in the salmonid gills, designated as the interbranchial lymphoid tissue (ILT) (19). In contrast to the dispersed organization typical of the GIALT, the ILT consists of dense clusters of T cells and antigen presenting cells located at the base of gill filaments (19). This spatial compartmentalization is consistent with a role for the ILT as a localized immune induction site, although its precise immune role is still not clearly defined.

Although different studies have demonstrated the immune regulation of the immune elements present in the gills to a pathogenic encounter or an antigenic stimulation (20–23), there is no information to date regarding how gill immune elements respond to a secondary stimulation, or whether immune memory responses are locally organized. To provide further insights into this matter, in the current study, we have analyzed the primary and secondary immune responses of the rainbow trout gills using *Lactococcus petauri* as a model pathogen. This bacterium is nowadays the main species responsible for lactococcosis outbreaks in rainbow trout, often exhibiting high mortality rates along with great economic losses (24). Lactococcosis is an important systemic disease primarily affecting internal organs such as the kidney, liver and spleen, often associated with septicemia, hemorrhages, yet necrosis and hemorrhages in the gills are also often observed (25).

To investigate the local responses elicited, after a first infection and subsequent reinfection by bath with *L. petauri*, we have investigated the transcriptomic profile of the gills using RNA sequencing (RNAseq), thereby identifying expression changes associated with either primary and secondary immune responses. Additionally, we performed a repertoire analysis by next generation sequencing (NGS) of IgM and IgT to evaluate changes in mucosal BCR diversity in the gills. Finally, we have carried out ELISpot assays to evaluate the number of cells secreting total and *L. petauri*-specific IgM among gill leukocytes as well as specific IgM titers in serum. Our results indicate that previously exposed fish mount a more efficient and controlled secondary response upon reinfection with *L. petauri*, reflected by decreased transcription of genes related to inflammation and cytokine responses, but a higher transcription of genes related with B and T cell function. Additionally, it was the group infected twice that had the higher number of specific IgM-secreting cells in gills and specific IgM titers in serum. These results shed light into secondary immune responses in mucosal surfaces, an outmost important issue for the effective optimization of mucosal vaccines for use in aquaculture.

## 2 Methods

### 2.1 Fish

Rainbow trout (*Oncorhynchus mykiss*) were obtained as fertilized eggs from a certified commercial supplier and reared at the Aquaculture Research Center of the Agrarian Technological Institute of Castile and León (CIA-ITACyL). Eggs were incubated and larvae reared under standard conditions until fish reached the target body weight to begin the experiment. Throughout the rearing period, fish were maintained at 15 °C in a recirculating aquaculture system with continuous aeration under a 12 h light: 12 h dark photoperiod and were fed once daily with a commercial trout diet (Skretting AI, Stavanger, Norway) at 3% of body weight.

When fish reached the average size of 43.1 g, they were transferred to the infection facility and, at this point, the water temperature was gradually increased from 15 °C to 18 °C at approximately 0.5 °C per day, reaching the desired temperature two weeks before performing the bacterial challenge. All the experiments described comply with the Guidelines of the European Union Council (2012/63EU) and the Spanish law (RD 53/2013) for the use of laboratory animals. They were approved by the INIA Ethics Committee (PROEX 065.3/21).

### 2.2 Bacteria

*Lactococcus petauri* isolates were obtained from Skretting Spain in a Brain Heart Agar (BHA) plate containing individual colonies. For each infection experiment, single bacterial colonies were cultured into 200 mL of Tryptic Soy Broth (TSB) (Pronadisa, Spain) and incubated at 30 °C for 18 h, with gentle agitation (100 rpm) under aerobic conditions. The concentration of *L. petauri* was determined using the endpoint dilution method previously described (26). The stock of bacteria was then adjusted to 5 x 10^14^ CFU/mL.

### 2.3 Experimental design

**Figure 1:**
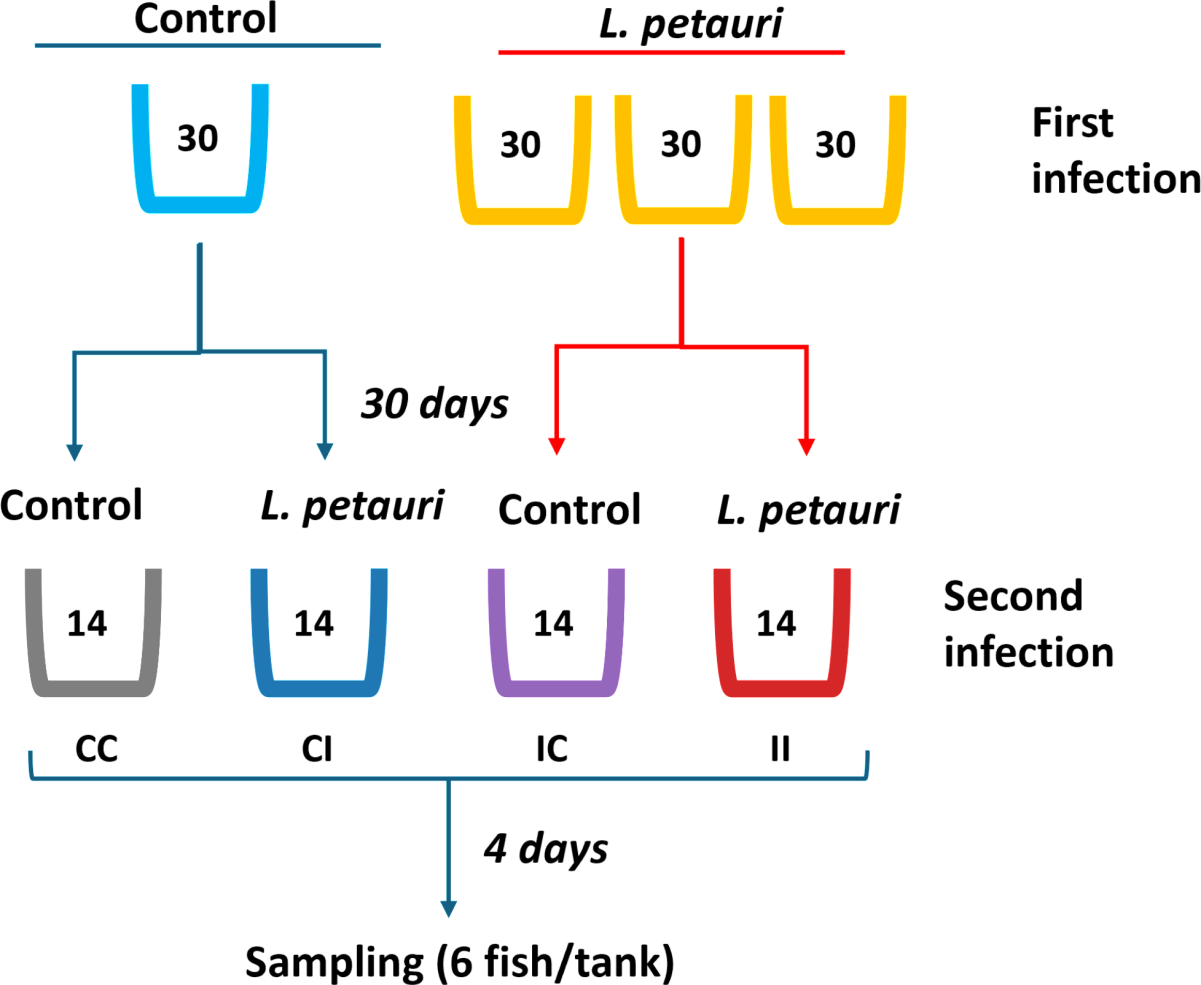
Schematic representation of the experimental design. During the first challenge, fish were either infected with *L. petauri* by bath or mock-infected (controls). After 30 days, survivor fish from the infected group and mock-infected fish were divided into two tanks, each of which were again infected or mock-infected. Thereby, fish that had not been infected in the first round were divided into either control-control (CC) and control-infected (CI), while fish that had survived the primary infection were divided into groups that were designated as infected-control (IC) and infected-infected (II) groups. Four days after the second infection, six fish in each of the four groups were sacrificed and blood and gill samples collected.

The infections with *L. petauri* were always performed by bath as schematized in **Figure 1**. Briefly, fish were collected from their tanks and placed into two aerated 15 L basins containing either *L. petauri* at 1 × 10^12^ CFU/mL or tryptic soy broth (TSB) alone (for mock-infected controls). After 2.5 h of immersion, fish were transferred back to their original 500 L tanks (one for the mock-infected control and three tanks for the infected fish). Fish were maintained at 18 °C for 30 days and during this period, mortalities were experienced in the infected (57% mean mortality) and none in the mock-infected group.

Following this period, a secondary challenge with the same bacterial strain was conducted using the same protocol. For this, survivors from the initially infected group were divided into two tanks of 14 fish each, one being re-infected with *L. petauri* and the other one mock-infected with TSB alone. In parallel, 14 fish from the previously uninfected control group were exposed to *L. petauri* for the first time, following the same procedure. The remaining 14 control fish were mock-infected with TSB alone. At day 4 after this last infection, 6 fish were sampled from each group. This design yielded four experimental groups: fish never exposed to the bacteria across either infection round (CC); fish exposed only in the second round, having encountered the bacteria once, 4 days before sampling (CI); fish exposed only in the first round, having encountered the bacteria once, 34 days before sampling (IC); and fish exposed in both rounds, having survived a primary infection before being re-challenged (II).

### 2.4 Tissue sampling and leukocyte isolation

At each sampling point, 6 fish from each group were euthanized by benzocaine (Sigma-Aldrich) overdose (250 mg/L). Prior to tissue extraction, and to reduce blood content in the tissues, fish were bled from the caudal vein with a heparinized needle. This blood was used for serum extraction following the protocol previously established (27) and the resulting samples stored at -80 °C until use.

A small piece of gills containing the ILT was removed and placed in RNAlater (Sigma-Aldrich) for posterior RNA extraction. The rest of the gills were then removed and leukocytes isolated for posterior ELISpot analysis. For this, the gills were first washed in a Petri dish with 5 mL of Leibovitz’s medium (L-15) supplemented with 100 IU/mL Penicillin-Streptomycin (P/S), 2% fetal calf serum (FCS) and 10 U/mL heparin. Single-cell suspensions were obtained by pushing the gill tissue through a 100µm nylon cell strainer (BD Biosciences). Gill samples were then washed by centrifugation (400 x *g* for 15 min) to eliminate excessive debris. Cell suspensions were transferred onto 30/51% discontinuous Percoll (GE Healthcare) density gradients and centrifuged at 400 x *g* for 30 min at 4°C, without brake. Cells in the interface, corresponding to leukocytes, were collected and washed in L-15 containing P/S and 5% FCS. The viable cell concentration was determined by Trypan Blue (Sigma-Aldrich) exclusion and cells were adjusted to a final concentration of 2 x 10^6^ cells/mL.

### 2.5 RNA extraction

The gill samples were removed from RNAlater and total RNA was extracted from the tissue using TRIzol Reagent (Fisher Scientific), following the manufacturer’s instructions and after homogenizing the tissue in TRIzol with a QIAGEN TissueLyser II. RNA was then quantified using a NanoDrop 1000 Spectrophotometer (Thermo Fisher Scientific). RNA integrity was assessed with a 2100 bioanalyzer using the Eukaryote Total RNA Nano assay (Agilent).

### 2.6 RNAseq

Gill RNA samples from four individuals within each experimental group (CC, IC, CI and II) were sent to Dreamgenics facilities (Oviedo, Spain) to perform an RNAseq transcriptomic study. In this case, total RNA (500 ng) was used for library preparation using the MGIEasy Fast RNA Library kit with polyA capture on the MGISP-100 platform (CE-IVD). The RNA libraries were sequenced on the DNBSEQ-G400 platform (CE-IVD) to produce 150 bp paired-end reads (2 x 150 bp). Quality control of the raw paired-end reads was performed using FASTP (28) and low-quality bases at the 3’ ends of reads were trimmed using a Phred quality threshold of Q20. Reads were aligned with HISAT2 (29) to the *Oncorhynchus mykiss* reference genome assembly USDA_OmykA1_1 (accession GCF_013265735.2) (Arlee genome) obtained from NCBI. Gene annotation was retrieved from the corresponding NCBI RefSeq GTF file associated with this assembly. Gene expression quantification was performed using featureCounts from the Subread package (30). Read counts were summarized at gene level by assigning reads to exonic regions and grouping them by gene_id attribute from the GTF annotation. The expression matrix was imported into R (v 4.4.3) for differential gene expression analysis using DESeq2 package (v 1.48.2) (31).

### 2.7 IgM and IgT repertoire analysis

An IgM and IgT repertoire analysis was performed in the four gill samples from each group selected previously for the RNAseq (CC, IC, CI and II). Library construction was performed following a custom protocol. For cDNA synthesis, 700 ng of total RNA were used in a reaction including an Oligo-dT primer (**Supplementary Table 1**) and a template switch oligo (TSO) incorporating a unique molecular identifier (UMI), that hybridizes to untemplated C nucleotides added by the reverse transcriptase during reverse transcription. The cDNA synthesis was carried out with SMARTScribe Reverse Transcriptase (Takara Bio) at 42°C for 90 min. Thereafter, each reaction was treated with 1 µL of uracil DNA glycosylase (5 U/µL, New England Biolabs) at 37°C for 40 min. The resulting cDNA was purified with NucleoSpin Gel and PCR Clean-up (Macherey-Nagel) and eluted in 20 µL. Two rounds of PCR were performed to enrich the 5′ end of IgM and IgT encoding transcripts followed by a third PCR for dual indexation, using in all reactions the Q5 High-Fidelity DNA polymerase (New England Biolabs). For the first PCR, 1 µL of purified cDNA was used as a template in a reaction with the “Target_Enrichment_FW1” (**Supplementary Table 1**), and the primers R1 of each IgH (located in the constant region of IgM or IgT) as a reverse, at a final concentration of 0.2 µM each. The PCR program was carried out with 20 cycles (95°C for 10 s, 60°C for 20 s and 72°C for 40 s). The resulting PCR product was purified using NucleoSpin Gel and PCR Clean-up (Macherey-Nagel) and eluted in 25 µL. For the second PCR, 1 µL of the purified PCR product was used as a template in a reaction containing the “Target_Enrichment_FW2” primer (**Supplementary Table 1**), which is partially complementary to Target_Enrichment_FW1, and the R2 primer specific of each IgH, located within the constant region of the corresponding IgH and that includes an additional tail corresponding with first part of P7 Illumina adapter. The PCR program was performed with 18 cycles (95°C for 10 s, 60°C for 20 s and 72°C for 40 s) in a final reaction volume of 25 µL. The PCR product was purified with NucleoSpin Gel and PCR clean-up. For the last PCR, 1 µL of purified product from the second PCR was used for sample indexing. Two sample-specific 8 nt indices were incorporated, one on each side of the amplicons, together with the final region of P5 and P7 Illumina adaptors. The sample-specific primers were used at a final concentration of 0.2 µM in a final reaction volume of 25 µL. The PCR program was performed for 12 cycles (95°C for 10 s, 54°C for 20 s and 72°C for 40 s). The PCR products were then visualized on a 1% agarose gel to verify the correct amplicon size and 10 µL of each sample were mixed together. Subsequently, two purification steps were performed: first one using NucleoSpin Gel and PCR Clean-up with NTI buffer diluted 1/3 an eluted in 25 µL. This product was then size selected using SPRIselect beads (Beckman Coulter) at a concentration of 0.6x. The quality of the final library was evaluated with Agilent Agilent TapeStation and quantified with the QubitTM dsDNA HS Assay Kit (Invitrogen, Life Technologies). Libraries were then sequenced on a NextSeq 2000 platform (Illumina) using NextSeqTM 2000 P2X reagents (600 cycles).

Raw paired-end reads were first merged using the PEAR software with default settings (32) to reconstruct full length amplicon sequences. Demultiplexing of the merged reads was performed using the FASTX-Barcode-Splitter tool from FASTX-Toolkit. To ensure correct isotype assignment during demultiplexing, one mismatch and a partial overlap of one nucleotide were allowed. The first 20 nt of the reverse primers used in the PCRs were employed as barcodes to identify of 3`ends corresponding to the constant gene of each Ig. UMI processing and consensus sequence generation were performed using MIGEC software. This tool groups reads sharing identical UMIs and generates consensus sequences (MIGs) using the first read as a template. MIGs with less than 5 reads were discarded. Finally, the assembled sequences were trimmed 14 nt at the beginning and 20 nt at the end to remove residual sequences derived from the template-switching oligo (TSO) and PCR primers. The resulting IgM and IgT sequences were aligned to the *O. mykiss* reference data available in the international IMGT database (33) using the IMGT/HighV-QUEST tool (v1.9.5) with the Arlee genome germline reference set.

### 2.8 ELISpot

The number of total and specific IgM-secreting cells in isolated gill leukocytes obtained from the sampled fish was determined using ELISpot. For this, ELISpot plates (Millipore) were activated with 70% ethanol and coated with an anti-IgM mAb (clone 1.14) (34) at 2 μg/mL in PBS or with previously inactivated (60°C for 1 h) *L. petauri* (5 x 10^13^ CFU/mL) and incubated overnight (ON) in continuous agitation at 4°C. Before adding the leukocytes, wells were washed 5 times with PBS (Sigma Aldrich) and incubated with 2% bovine serum albumin (BSA, Sigma Aldrich) in PBS for 2 h at room temperature (RT), to block non-specific binding. Gill leukocytes were then added to the wells in duplicate at a concentration of 1 x 10^5^ cells per well, immediately after isolation. After 24 h of incubation at 20°C, cells were washed five times with PBS and plates blocked for 1 h at RT with 2% BSA in PBS. After the blocking step, biotinylated anti-IgM mAb (clone 1.14) was added to the plates at 1 μg/mL and incubated for 1 h at RT in agitation. Following additional washing steps (five times in PBS), the plates were developed using streptavidin-HRP (Thermo Scientific) at RT for 1 h in continuous agitation, washed again five times with PBS and incubated with 3-amino-9-ethylcarbazole (Sigma Aldrich) for 30 min at RT in the dark. The substrate reaction was stopped by washing the plates with water. Once the membranes had dried, they were digitally scanned and the number of spots in each well was determined using an AID iSpot Reader System (Autoimmun Diagnostika GMBH).

### 2.9 ELISA

The levels of specific IgM were also evaluated by ELISA in the sera of all sampled fish. To conduct the ELISA, 96-well ELISA plates were coated ON with previously inactivated (60°C for 1 h) *L. petauri* (5 x 10^13^ CFU/mL) diluted in 0.05 M carbonate buffer at pH 9.7. After 3 washes in 0.05% Tween-20 PBS (PBST), wells were blocked with 100 μl of 1% BSA in PBST for 1 h at RT. Plates were then washed 3 times with PBST and thereafter serum samples were diluted 1:100 in PBS containing 1% BSA and added to the plates. Samples were incubated 1 h at RT and washed 3 times in PBST. Thereafter, 50 μl of biotinylated anti-trout IgM mAb (1 μg/mL) diluted in blocking buffer were added to the wells. After 1 h of incubation at RT, the wells were washed 3 times with PBST and then incubated with 50 μL of Streptavidin-HRP (diluted 1:1000 in PBS supplemented with 1% BSA) for 1 h at RT. Wells were washed again 3 times and then 50 μL of OPD (O-Phenylenediamine Dihydrochloride) (Sigma) added (1 μg/mL). The reaction was stopped by adding 50 μL of 2.5 M H_2_SO_4_ and absorbance at 492 nm measured in a FLUO Star Omega Microplate Reader (BMG Labtech). Positive and negative controls were included in all the plates, and consisted in sera from infected and non-infected rainbow trout from another experiment, respectively. Wells with serum but with no biotinylated anti-trout IgM mAb were also included.

### 2.10 Statistical analysis

For the RNAseq, differential gene expression was analyzed using DESeq2 package (v 1.48.2), using a negative binomial distribution (31). The Wald test was applied to identify genes with significant differences between groups and *p* values were adjusted for multiple testing using the Benjamini-Hochberg method (FDR < 0.05). Pairwise comparisons between groups were performed. Only genes with an adjusted *p* value < 0.05 and a Log2 fold change (FC) ≥ |1| were considered differentially expressed.

For the IgM and IgT repertoire data, normality was first assessed using the Shapiro-Wilk test. Data with normal distribution were analyzed using ANOVA to evaluate overall differences among groups, followed by Tukey’s HSD *post-hoc* test for pairwise comparisons. Data with non-normal distribution were analyzed using the Kruskal-Wallis test to evaluate overall differences among groups, followed by Dunn test for pairwise comparisons. To check the distribution of the CDR3 spectratyping, a Shapiro-Wilk test was performed. To assess differences in CDR3 length distributions among groups, a permutational multivariate analysis of variance (PERMANOVA) was performed using Bray-Curtis dissimilarity implemented in the R package *vegan* (v 2.7.2). Distance matrices were calculated from the relative frequencies of each CDR3 length per sample. Statistical significance was evaluated using 999 permutations. Homogeneity of multivariate dispersions among groups was assessed using the betadisper test. Pairwise PERMANOVA comparisons were performed between groups and *p* values were adjusted for multiple testing using Benjamini-Hochberg method. Data were presented as mean + SD and significance between means was stablished at *p* value ≤ 0.05, indicating in some cases different degrees of significance (* *p* ≤ 0.05, ** *p* ≤ 0.01 and *** *p* ≤ 0.005). In the case of VDJ configuration, differences within each isotype were represented using letters, with values that do not share any letter considered significantly different.

The data from ELISA and ELISpot were analyzed and handled with GraphPad Software (GraphPad Prism v8.0.1, La Jolla California, USA). Prior to analysis, data were checked for normality using Shapiro‐Wilk test. To compare the levels of specific IgM analyzed by ELISA and the number of total and specific IgM-secreting cells analyzed by ELISpot among the different groups, an ANOVA was performed followed by a Tukey’s HSD post‐hoc test when data were normally distributed, or evaluated by a non‐parametric Kruskal‐Wallis test when they were not normal.

## 3 Results

### 3.1 Transcriptional response of the gills to *L. petauri* primary and secondary infections

**Figure 2:**
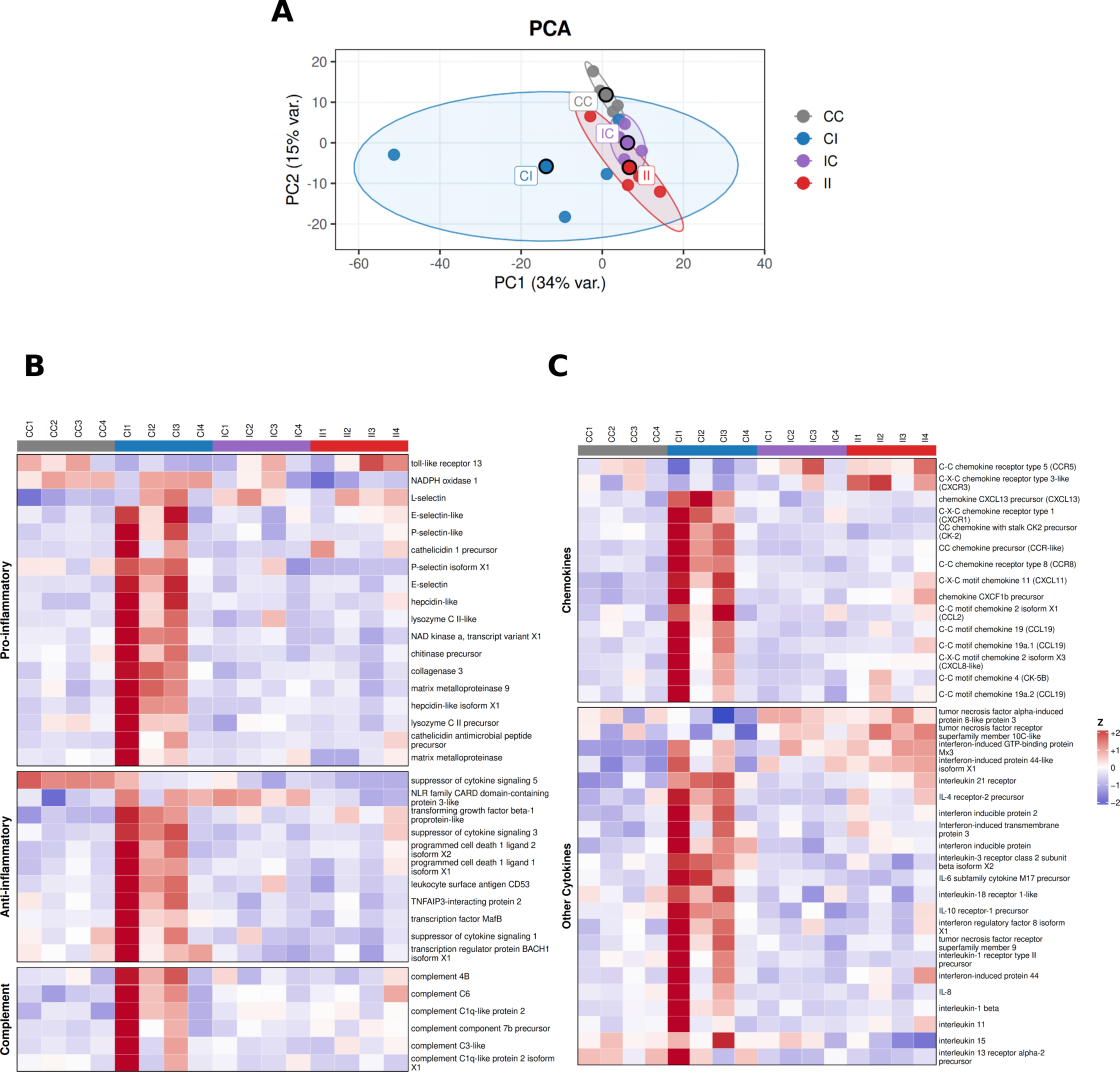
Transcriptional response of the gills to *L. petauri* primary and secondary infections. Four individuals in each group were selected to perform an RNAseq analysis in the gills. Principal component analysis (PCA) based on normalized gene expression data **(A)** and heatmaps showing differentially expressed genes (DEGs) in each experimental group (*p* adj < 0.05; |log2FC| ≥ 1) for genes related to inflammation and complement **(B)** and chemokines and other cytokines as well as their receptors **(C)**. Expression values are represented as Z-scores, calculated for each gene by subtracting the mean and dividing by the standard deviation across all samples. Red indicates gene expression above the mean, blue indicates expression below the mean and gray indicates expression close to the mean.

To analyze the immune response of the gills to a primary and a secondary infection with *L. petauri*, we selected four sampled individuals from CC, CI, IC and II groups to undertake an RNAseq analysis. The principal component analysis (PCA) of global gene expression in gill samples (**Figure 2A**) revealed clear groupings reflecting infection status and timing. Control fish (CC), as well as fish infected once in the first round (IC) and those infected twice (II), showed relatively tight clustering, indicating more consistent expression profiles within these groups. In contrast, fish infected only in the second round (CI) exhibited a much greater dispersion across PC2, reflecting higher variability in gene expression and suggesting a differentiated transcriptional response to recent infection in fish that had not been previously exposed to the bacteria.

**Figure 3:**
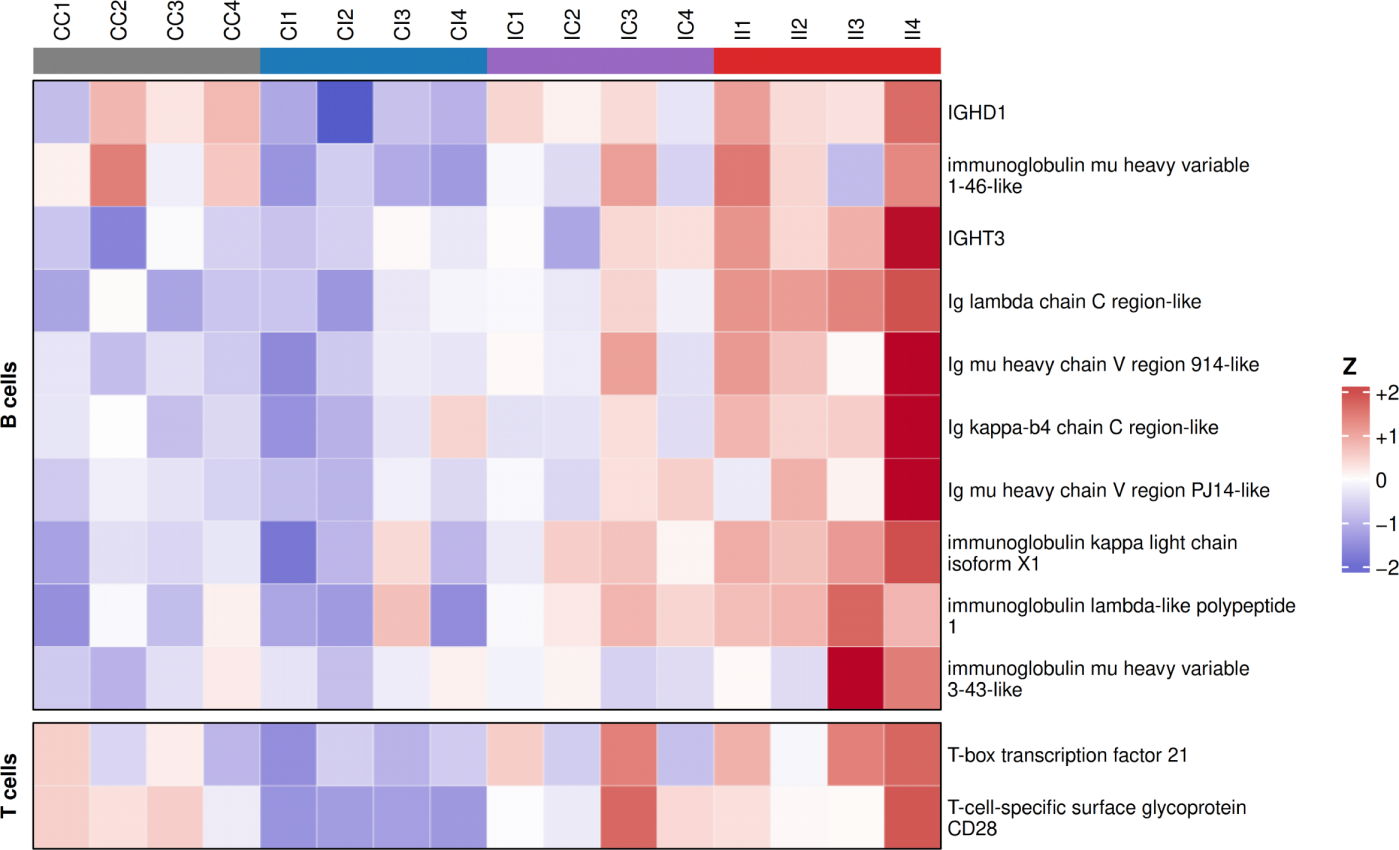
Heatmap of differentially expressed adaptive immunity genes in gill samples. Heatmap showing DEGs (*p* adj < 0.05; |log2FC| ≥ 1) in each experimental group across individual gill samples for adaptive genes related to either B or T cell function. Expression values are represented as Z-scores, calculated for each gene by subtracting the mean and dividing by the standard deviation across all samples. Red indicates gene expression above the mean, blue indicates expression below the mean and gray indicates expression close to the mean.

We then analyzed the genes that were differentially expressed genes (DEGs) with *p* values <0.05 and log2 fold change > |1| (**Supplementary Table 2**). The total number of DEGs identified in each pairwise comparison was as follows: CC vs CI, 611 genes; CC vs IC, 361 genes; CC vs II, 731 genes; CI vs IC, 477 genes; CI vs II, 574 genes; IC vs II, 32 genes (**Supplementary Table 2**). These genes were grouped according to their main immunological functions, and from all of these DEGs we made special focus on genes related to inflammation, such as pro-inflammatory mediators, anti-inflammatory regulators, complement components (**Figure 2B**), chemokines and other cytokines (**Figure 2C**) and genes related to adaptive immunity, including both B and T cells (**Figure 3**).

We first examined genes coding for pro-inflammatory and anti-inflammatory factors and complement components. In this case, the fish that had only received the bacteria in the second round (CI) and were therefore recently infected at the time of sampling, had levels of transcription of most of these genes significantly higher than those of all other groups (**Figure 2B**). Within pro inflammatory mediators, genes up-regulated in the CI group when compared to other groups included those coding for NADPH oxidase 1, lysozyme C, cathelicidins and hepcidin-like peptides. Interestingly, within this group of genes, *tlr13* transcription was differently regulated across groups, with higher mRNA levels in the II group, higher than those reached in the CI groups (**Figure 2B**). The response of genes cataloged as anti-inflammatory was similar to that of the previous group, with most genes being transcribed at significantly higher levels of expression in the CI group when compared to the other groups (**Figure 2B**). This group included genes coding for SOCS3, SOCS1, TNFAIP3, transforming growth factor β (TGF-β), PD-L1-like molecules and the transcription factor MafB (**Figure 2B**). Again, there was one exception within this category, since the gene coding for suppressor of cytokine signaling 5 was expressed at significantly higher levels in the control fish (CC) when compared to other groups (**Figure 2B**). The response of complement components was similar to that of pro-inflammatory and anti-inflammatory factors, with highest expression levels in the CI group (**Figure 2B**). The complement factors differentially regulated included 4B and 7B, C1, C3 and C6 (**Figure 2B**).

We also analyzed in more detail within the DEGs, those coding for chemokines or chemokine receptors or other cytokines and their receptors (**Figure 2C**). Among the genes differentially transcribed we found chemokine-coding genes such as *cxcl8* (coding for IL8), *ccl19/ccl19a, cxcl13, ck2 and ck5b* with higher expression levels in the CI group when compared to other groups (**Figure 2C**). Concerning receptors genes such as *ccr5* and *cxcr3* had higher mRNA levels in the II group, whereas *cxcr1* and *ccr8* had higher mRNA levels in the CI group (**Figure 2C**). As for other cytokines classes, several genes with antiviral and immunoregulatory functions were also differentially expressed across treatments. The CI group showed a strong induction of genes coding for proteins related to interferon (IFN) activity such as IRF8, IFN inducible protein 2 and IFN-induced transmembrane protein 3 (**Figure 2C**). Different interleukins were also differentially more expressed in the CI group compared to the other groups, including IL1β, IL8, IL11 and IL15 (**Figure 2C**). Cytokine receptors transcriptionally up-regulated in the CI group when compared to other groups included IL-21R, IL-4R, IL18-R, IL10R, TNFRSF9 (tumor necrosis factor receptor superfamily member 9) and IL-13R (**Figure 2C**). Also in this case, some exceptions were identified, with genes significantly up-regulated in the II group compared to the others including those coding for TNFα-induced protein 8-like protein, a TNFRSF10C and Mx3 (**Figure 2C**).

Finally, we selected, among the DEGs, those related to the functionality of adaptive cells. Concerning B cell function, DEGs included *igdh1*, multiple IgM variable heavy chain segments, *ight3*, and κ and γ-like light chains, which were all differentially expressed, with higher mRNA levels in the II group when compared to the rest of the groups (**Figure 3**). The genes expressed at significantly higher levels in the II group also included two genes associated with T cell function, namely *tbx21* and *cd28* (**Figure 3**).

### 3.2 IgM and IgT VDJ rearrangements in the gills

**Figure 4:**
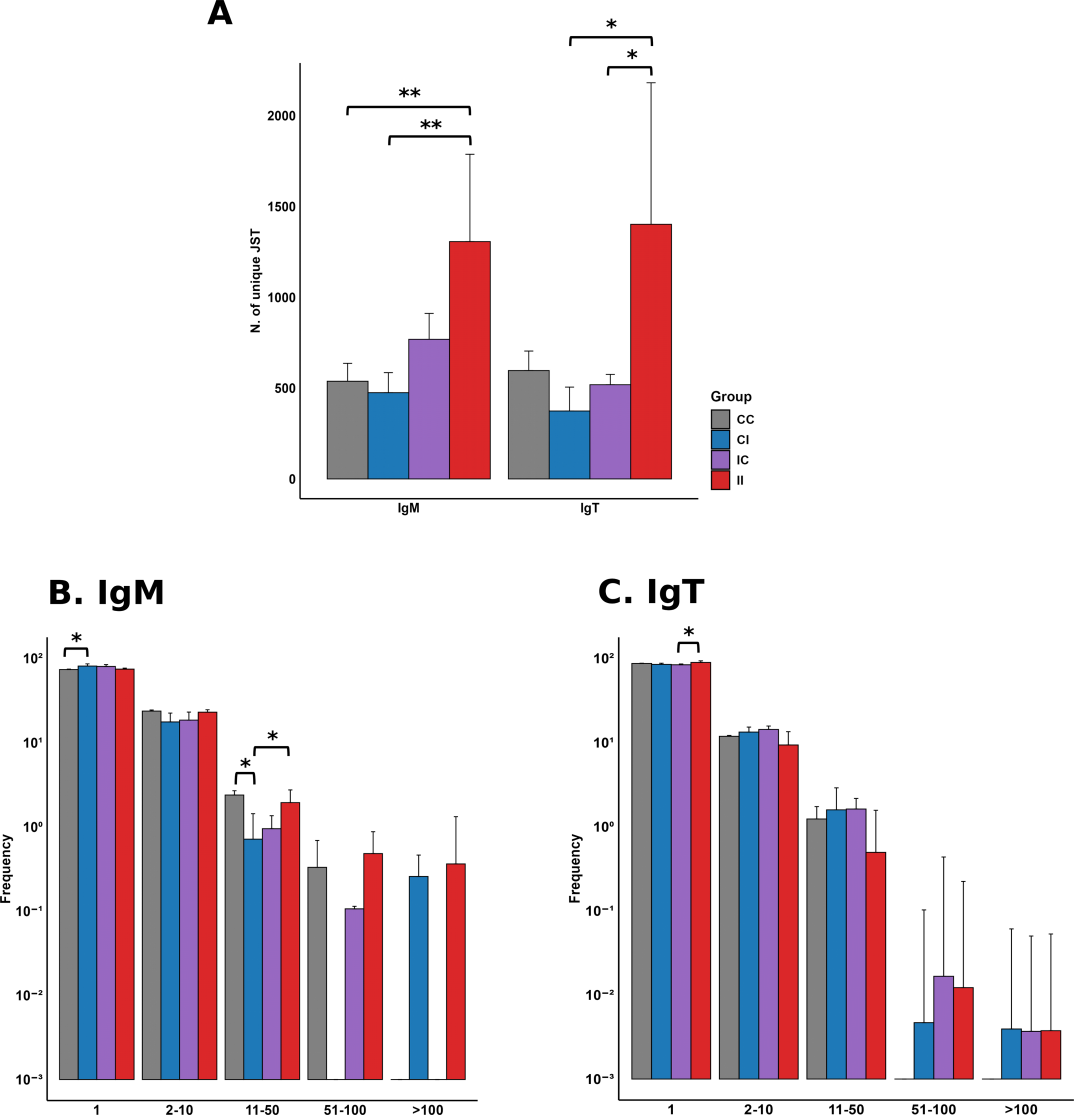
JST distribution of Ig genes in the gills in the different experimental groups. IgM and IgT sequences were grouped by unique junction sequence types (JSTs). Each unique JST is defined as V_H_DJ_H_ rearrangement together with a specific CDR3 amino acid sequence. **(A)** Bar plot showing the number of unique JST junctions split by IgH chain (mean + SD, n = 4). **(B, C)** Grouped clonal size distribution for IgM **(B)** and IgT **(C)** across the different groups. Bar charts show the relative frequency of JSTs observed n times in each group (mean + SD, n = 4). Significance between means was stablished at *p* value < 0.05, indicating in some cases different degrees of significance (*p ≤ 0.05 and **p ≤ 0.01).

To characterize how a primary and a secondary infection with a bacterial pathogen affected the mucosal BCR diversity in gills, an Ig repertoire analysis was performed in the four individuals from the different groups that had been selected for the RNAseq analysis. We focused on IgM and IgT isotypes because IgD transcripts did not appear specifically regulated in the II group in the RNAseq analysis. Sequences were first grouped into unique JSTs, defined as a V_H_DJ_H_ rearrangement and their associated CDR3 amino acid sequence. Substantial differences in the number of unique JST were observed among groups (**Figure 4A**). For both IgM and IgT isotypes, the highest number of unique JSTs was detected in the re-infected group (II). In the case of IgM, the number of unique JSTs in the II group was significantly higher than that of the control (CC) and the CI groups (**Figure 4A**). In the case of IgT, the II group showed a significantly higher number of unique JST than the CI and IC groups (**Figure 4A**).

To get an indication of how many expanded IgM^+^ and IgT^+^ B cells clones were in the gills of the different individuals, the clonal size distribution of IgM and IgT JSTs was then analyzed (**Figure 4B, C**). It is generally accepted that JSTs detected less than 3–5 times in a given tissue correspond to non-expanded antigen-naive B cells, whereas JSTs detected more times reflect expanded antigen-experienced B cell clones, including antibody-secreting cells (35). In the case of IgM, the number of JSTs only found one time was slightly higher in the CI group when compared to the CC group (**Figure 4B**). Some significant differences in the frequencies of sequences repeated 11 to 50 times were also observed, being higher in the CC group when compared to the CI, and higher in the II group when compared to CI (**Figure 4B**). Although the frequency of sequences repeated more than 100 times was higher in the CI and II groups, the mean value was not significantly different than other groups due to a very high individual variability (**Figure 4B**). In the case of IgT, the only significant difference among groups was in the number of sequences present only once, being significantly higher in the II group when compared to the CI group (**Figure 4C**). Although the sequences repeated more than 51 times where higher in the CI, IC and II groups than in the CC, the differences were not significant, again due to high individual variability (**Figure 4C**).

**Figure 5:**
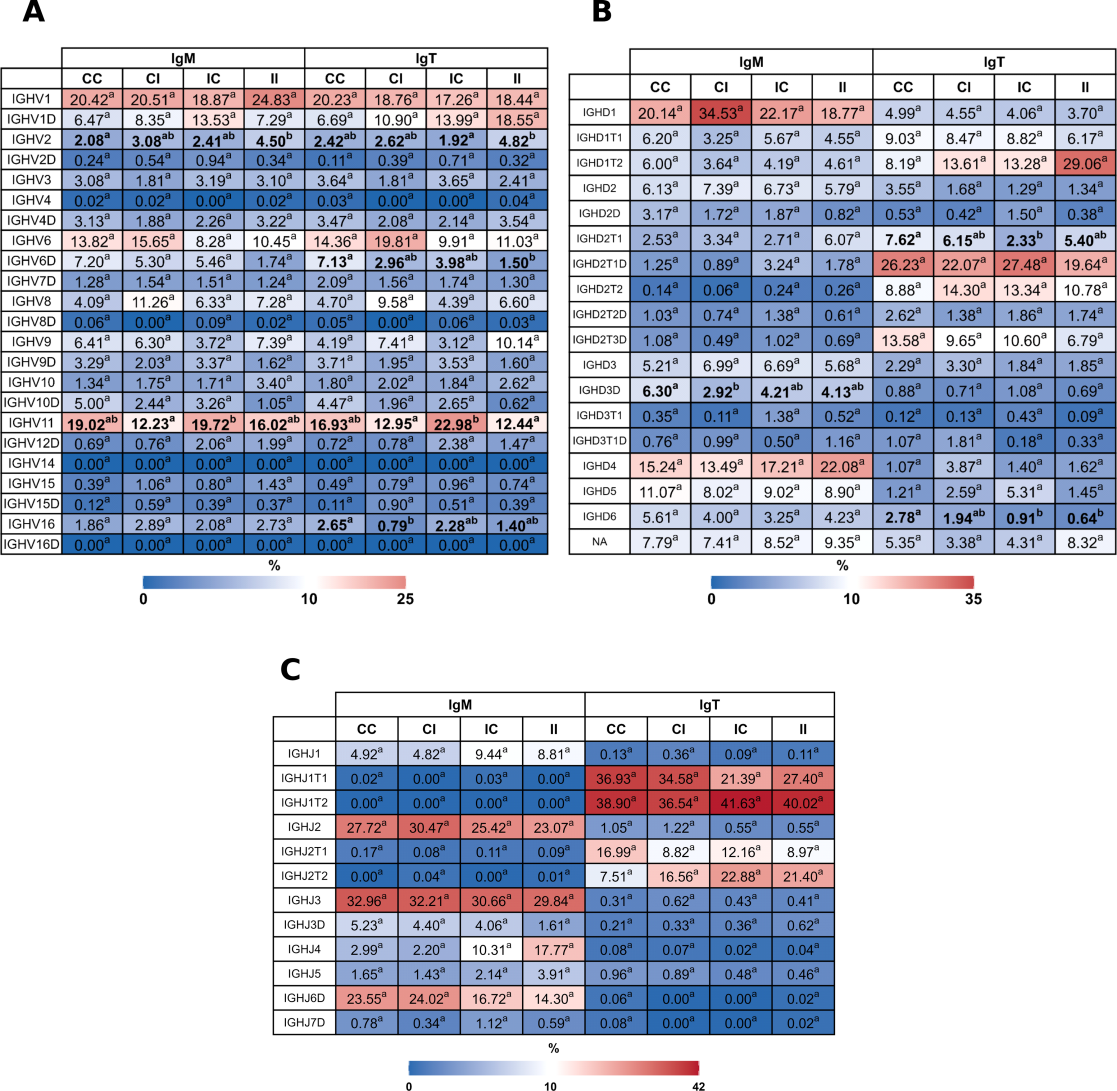
IgM and IgT V_H_DJ_H_ configuration in the gills within the different experimental groups. **(A–C)** Heatmap representations of usage of differential germline **(A)** V_H_, **(B)** D and **(C) ** J_H_ genes. Values represent the relative frequency of use of each gene per IgH chain. Higher values are shown in red, lower values are shown in blue. Different lowercase letters indicate significant differences among groups (*p* ≤ 0.05). These results are highlighted in bold.

We also analyzed the IgH V_H_DJ_H_ family usage for IgM and IgT. Overall, V_H_ gene usage was similar for both isotypes and across the different experimental groups with small non-significant variations (**Figure 5A**). IgM and IgT showed a preferential usage of IGHV1, IGHV6 and IGHV11 (**Figure 5A**). Some significant differences were found among groups in the case of IGHV11 usage for both IgM and IgT, with percentages in the IC significantly higher than those of the CI group (for IgM) and higher than those of the CI and the II groups for IgT (**Figure 5A**).

As expected, given that IgT has its own D segments, the use of D segments differed for IgM and IgT (**Figure 5B**). In this case, IgM preferentially used IGHD1 followed by IGHD4 and IGHD5, which are segments close to the IgM/D constant regions (**Figure 5B**). However, no significant differences were found in the usage of these and other segments across experimental groups, with one exception (**Figure 5B**). Hence, the usage of the IGHD3D segment was significantly lower in the CI group than in the CC group (**Figure 5B**). In contrast, IgT preferentially used D segments closely associated with the IgT constant regions, including IGHD2T1D, IGHD2T2, IGHD1T2 and IGHD2T3D (**Figure 5B**). Although only a few significant differences were found in the percentage of usage across groups, the II group showed a higher usage of IGHD1T2 compared to that of other groups, albeit with large individual variability (~29.06%) (**Figure 5B**). Although further away from IgT constant sequences, some of the sequences detected in the gills contained segment IGHD6 and, in this case, its use was significantly reduced in the IC and II groups when compared to the CC group (**Figure 5B**).

As occurs with the D segment, IgM preferentially used J_H_ segments close to the IgM/D constant regions and IgT those closer to its constant regions (**Figure 5C**). Hence, IgM preferentially used IGHJ3, followed by IGHJ2 and IGHJ6D (**Figure 5C**). However, no significant differences in segment usage were detected across groups (**Figure 5C**). In the case of IgT, IGHJ1T2 was the most J_H_ segment used, followed by IGHJ1T1, IGHJ2T2 and IGHJ2T1. Consistently, no statistical differences were detected in J_H_ segment usage among groups for IgT (**Figure 5C**).

### 3.3 CDR3 spectrotyping of IgM and IgT sequences in gills

**Figure 6:**
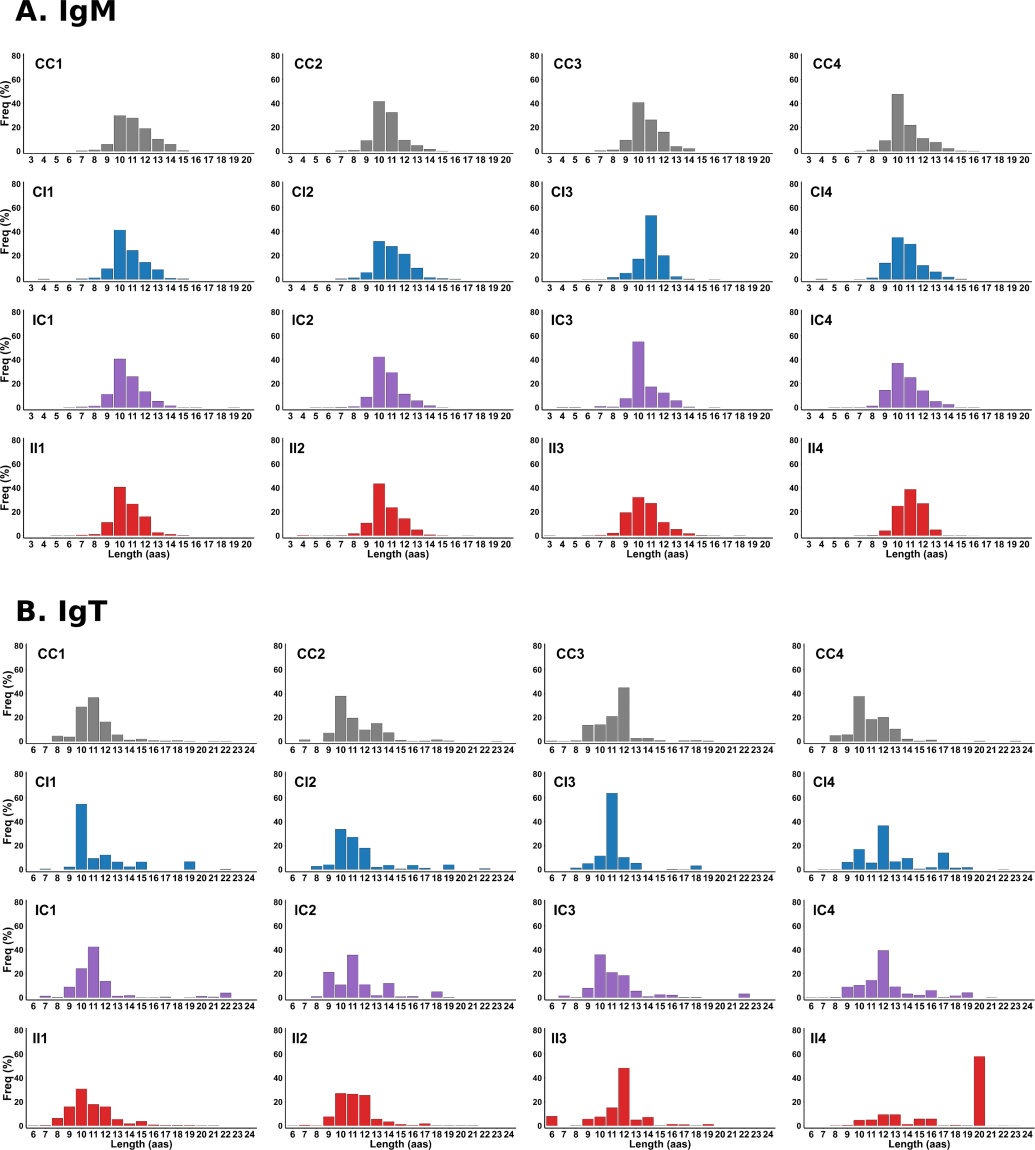
CDR3 length distribution of IgM and IgT repertoires in gill tissue across experimental groups. CDR3 spectrotyping of IgM **(A)** and IgT **(B)** repertoires in the gills in the different experimental groups. Bar plots show the relative frequency of each CDR3 length. Data distribution was evaluated using the Shapiro-Wilk test, determining that all graphs follow a non-normal distribution.

Because the length of the CDR3 region within the Ig variable region is altered throughout the differentiation process (36, 37), to further investigate whether IgM and IgT had experienced clonal selection in the different experimental groups, a spectratyping analysis of the CDR3 regions of the BCR was conducted. The aim was to evaluate the normality in the distribution of the frequency of the CDR3 regions length across groups, taking into account that a normal distribution of lengths indicates a non-disturbed naïve population, whereas deviations from normality indicates that there is clonal selection to some degree (36, 37). In the case of IgM (**Figure 6A**), the distributions of frequencies presented similar overall patterns across groups with none of them following a normal distribution. In every group and every individual fish, the most frequent CDR3 length was approximately 10 amino acids. As observed for IgM, IgT frequency distributions presented similar patterns across the groups and also deviated from a normal distribution (**Figure 6B**). In this case, the size of the CDR3 segments was slightly higher (**Figure 6B**). No significant differences were found in the distribution of CDR3 length frequencies among groups for either isotype.

### 3.4 *L. petauri*-specific IgM production

**Figure 7:**
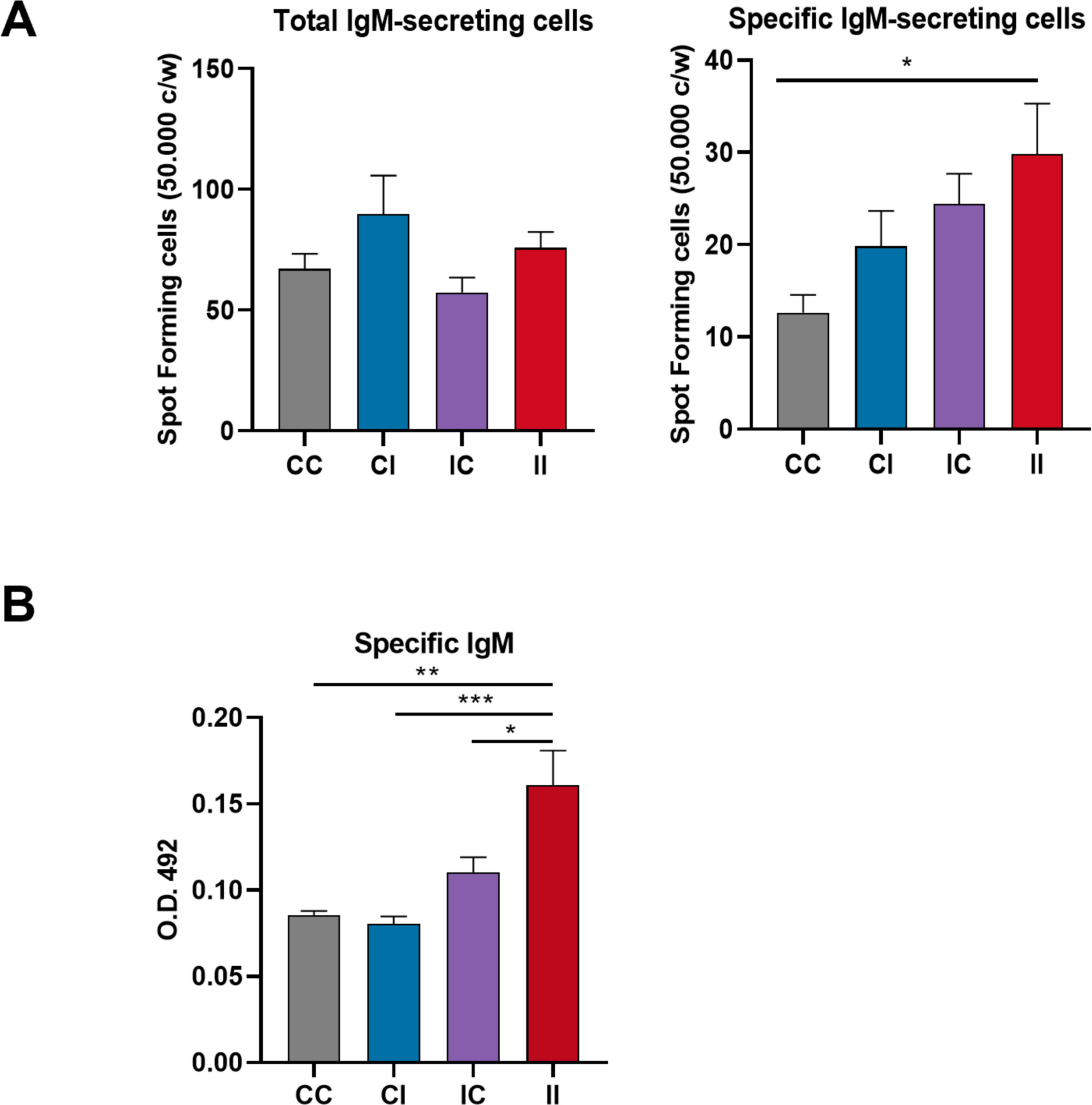
IgM secretion in the different experimental groups. Gill leukocytes were isolated from the sampled fish in each group and the number of cells secreting total or *L. petauri*-specific IgM among these populations determined by ELISpot, following the protocol described in the Materials and methods section **(A)**. The titers of *L. petauri*-specific IgM were also determined by ELISA in serum samples from the different experimental groups **(B)**. ELISA results are shown as mean absorbance values at 492 nm + SEM (n = 6), whereas ELISpot results constitute the number of cells secreting total or *L. petauri*-specific IgM per 5 × 10^4^ cells + SEM (n = 6). Asterisks denote significantly different values among groups as indicated (*p < 0.05, **p < 0.01 and ***p < 0.005).

To further investigate the secondary IgM responses of gill leukocytes, we determined the number of cells secreting total and *L. petauri*-specific IgM in gill leukocytes obtained from the different experimental groups. We found that, although no significant differences were detected in the number of total IgM-secreting cells, the number of specific IgM-secreting cells was significantly increased in the re-infected group (II) compared to the control group (CC) (**Figure 7A**; **Supplementary Figure 1**).

To confirm whether this specific secondary response was also taking place at systemic level, we also determined the levels of *L. petauri*-specific IgM in serum. In this case, specific IgM levels were significantly elevated in re-infected fish (II) compared to CC, CI and IC groups (**Figure 7B**).

## 4 Discussion

Although teleost fish are capable of mounting both innate and adaptive immune responses to antigen exposure, being this the basis of vaccination, whether secondary immune responses take place in fish in a similar way as in mammals is still a matter of debate. In mammals, when the immunized individuals see an antigen for the second time, the immune response elicited is much faster and stronger than the initial one, thanks to the presence of memory B and T cells (38). In fish, there is little research regarding the functionality of T cells and for this reason the presence of memory T cell responses has never been demonstrated in any fish species (39). For B cell responses, although the presence of cells with a memory-like profile has been reported in some species (40), and in some experiments slightly increased antibody responses are seen after re-stimulation (41, 42), there are plenty of examples in which this increased secondary is not clearly visualized (43–45). Furthermore, whether B cell memory responses are mediated by long-lived plasma cells that become re-activated, or whether it is a dedicated independent B cell population with a phenotype similar to mammalian memory B cells, is not fully resolved in fish yet (42), partly due to the high heterogeneity of memory B cell subsets also observed in mammals (46).

To better understand secondary responses in fish, some studies have investigated the transcriptional response of vaccinated animals upon a posterior challenge. In this sense, our group performed many years ago a study in which rainbow trout DNA vaccinated against viral hemorrhagic septicemia virus (VHSV) were posteriorly challenged with the live virus (47). The levels of transcription of a selection of immune genes were then analyzed in head kidney, spleen and blood by PCR. Surprisingly, most of the genes that seemed up-regulated in the vaccinated and challenged group to higher levels than those of the non-vaccinated and challenged group were genes associated with innate responses such as those coding for IL1β, type I IFN or Mx (47). These results have been supported by a very recent study from our group, in which we determined the transcriptional response of leukocytes obtained from VHSV DNA vaccinated trout when they were exposed *in vitro* to the virus. In this case, the results demonstrated that leukocytes from vaccinated fish had a superior capacity to produce innate antiviral cytokines in response to the virus, and also had higher transcription levels of *mda5*, a gene that encodes a virus-sensing innate receptor (48). Although both studies seem to question the organization of a true secondary immune response in DNA vaccinated fish, a similar study conducted by Pereiro and collaborators in turbot (*Scophthalmus maximus*) used RNAseq to determine the transcriptional response of head kidney in VHSV DNA vaccinated fish upon viral challenge, demonstrating a profile coherent with a classical secondary immune response (49). Specifically, they determined that the vaccinated animals had a profile consistent with activation of acquired immunity when challenged with the virus, absent in non-vaccinated animals that were more inclined to pro-inflammatory responses (49). However, this type of studies have never been undertaken in a teleost mucosal tissue. Thus, to date, among the many unknowns of how mucosal immune responses are orchestrated in fish, is whether memory responses are locally organized.

Our first approach was to use RNAseq to decipher the transcriptomic profile of the gills in fish exposed to the bacteria (*L. petauri*) twice, only one (at different times) or never at all. We decided to sample the fish at day 4 post-infection since this was a time point at which we had the highest transcriptional responses in the rainbow trout gills when bath infected with another bacterial pathogen (23). Although the kinetics and the effects of the different pathogens could be different, we decided, based on this, that day 4 was an adequate time point to detect early responses, but give the host enough time to mount a robust immune response. At this point, strong transcriptional effects were also found in the gills in response to a primary *L. petauri* infection. The genes that were significantly up-regulated in response to the bacteria when compared to non-infected fish included a wide range of factors involved in the regulation of inflammation, the complement system, as well as many cytokines/chemokines and their receptors. However, at this point, no genes directly related to adaptive immunity were significantly up-regulated by the primary infection when compared to controls. This pro-inflammatory response was not observed in the IC group that was exclusively exposed to *L. petauri* in the first infection step (34 days before sampling), indicating that the transcriptional effects of the bacteria on the gills are not maintained for such long periods. Interestingly, the secondary response to the bacteria in the II group was quite different from that of the CI group. In this case, a short-term exposure to the bacteria in fish that had previously survived the infection did not involve the up-regulation of the innate factors induced in the primary infection. In contrast and despite having sampled at an early time point post-infection, a wide range on genes related to adaptive immunity were up-regulated in the II group. Two of these genes are related to T cell activity (*tbox21* and *cd28*) but many correspond to different Ig chains, including light chains (kappa and lambda) or different heavy chains (IgM and IgT). The IgD chain seemed up-regulated in this II group but was also high in the CC group, therefore, in this case, IgD mRNA levels were not distinctively higher in the secondary infection. These results are coherent with a local memory response in which local lymphoid cells (especially B cells) seem better prepared to react to the bacteria locally, once they have been previously exposed to it. As a result, such a strong pro-inflammatory reaction as the one triggered in primary infections, does not seem necessary. Whether this is a result of systemic secondary responses organized in tissues such as spleen having a remote effect on the gills, cannot be completely ruled out, but they could also imply the presence of a local memory B (and maybe T) cell subset that stays in the mucosa and is reactivated upon re-infection. The presence of memory B cells in mucosal surfaces in mammals is well-known (38, 46), therefore it could be possible that this was the case also in fish.

Remarkably, two chemokine receptors (CCR5 and CXCR3) and one TLR (TLR13) followed a different pattern of transcription in the experimental groups than those of the other genes associated with an inflammatory or a cytokine response. The genes coding for these three proteins were expressed at significantly higher levels in the II group when compared to other groups. Both CCR5 and CXCR3 are key receptors responsible for the recruitment of T cells (50), with CXCR3 being more broadly expressed across T cell subsets (51) and CCR5 being preferentially expressed in Treg cells (52) and also in memory CD8^+^ T cells (53). Therefore, future studies aimed at investigating the role of CCR5 in secondary mucosal infections could provide us with interesting information regarding how T cells are regulated during this process and which T cell subsets are preferentially involved. TLR13, on the other hand, is a receptor expressed by innate immune cells in mice (but not in human) that specifically recognizes a conserved bacterial 23S ribosomal RNA sequence (54). The specific role of this receptor during secondary mucosal responses in fish also seems worthy of future investigations.

Given that both IgM and IgT transcripts were significantly up-regulated during the secondary response of the gills to *L. petauri*, our next step was to perform an extensive repertoire analysis of both isoforms. We thus found that not only the total number of IgM and IgT transcripts were higher in the II group (as determined in the RNAseq analysis), but also that of unique JSTs (unique transcript sequences). This, together with the fact that the number of sequences repeated more than 51 times was highly variable and not significantly different among groups, points to the secondary infection provoking an increased clonal diversity in the gills. The fact that very few specific V, D, J segments are differentially expanded across experimental groups further supports this hypothesis. The CDR3 spectrotyping analysis also pointed in this direction, since no clonal selection significantly different of that already present in control fish was evident in any of the infected groups. A previous study in which we compared the BCR repertoires in different tissues of unhandled rainbow trout had demonstrated that while the spleen CDR3 spectrotyping presents a clonal distribution, the profile in mucosal tissues is non-clonal, even in the absence of a specific challenge, probably due to selection mechanisms that normally take place in mucosal surfaces (17). In that same paper, the number of sequences repeated more than 10 times was higher in gills than in the spleen for both IgM and IgT (17), result that suggested that the gills contained a higher number of cells that had already started a differentiation towards antibody secreting cells (ASCs) and, therefore, produced more copies of one specific sequence. This was later confirmed by flow cytometry and ELISpot for IgM-secreting ASCs (9). Therefore, although teleost fish have been shown to be capable of undergoing clonal selection for both IgM and IgT in response to some antigens in systemic tissues (35), it seems that the nature of the B cell population in the gills that seems already antigen-experienced could be preventing this clonal selection from happening. Whether this is specific for *L. petauri* or occurs with other pathogens should nonetheless be investigated.

One of the limitations of our study is that although the sampling point (4 days after the second infection) seemed optimal for the RNAseq analysis, one might argue that it is too early to see changes in the BCR repertoire. Being this true and despite not having samples obtained at a later time point available from this experiment, it should be noted that at this time point we could detect an increased number of cells secreting *L. petauri*-specific IgM in gill leukocyte cultures and also higher *L. petauri*-specific IgM titers in serum from the II group. Therefore, if these increased titers would had been a result of clonal selection, we should have detected these changes in the BCR repertoires. Also, if gill B cells responded to *L. petauri* infection with clonal selection, we should have already seen some changes at repertoire level at least in the IC group (which was sampled 34 days after the bacterial exposure).

In the current study, we have sampled the gills to include both the lamellae and the ILT, however it would be interesting in the future to study secondary immune responses making a distinction between these two areas. To date, the precise role of the ILT is yet undefined, as studies have ruled out it being a primary immune tissue or having any connection to the thymus (55). Furthermore, its functionality as a secondary immune tissue is also doubted, mainly because, to date, no antigen-sampling capacities have been described for cells in the ILT in contrast to the rest of the gills (21, 56). Furthermore, antigen-exposure did not generate visible changes in Ig transcripts in this area or an expansion of the ILT (14), while decreases in size in response to bacterial challenge have been reported (57). Therefore, studying the precise response of this immune structure to a secondary pathogen exposure could be helpful to better clarify its immune role.

In conclusion, we have demonstrated that the gills respond differently to a primary than to a secondary exposure with a bacterial pathogen, revealing for the first time the presence of a mucosal secondary immune response. While the primary response of the gill is characterized by a pro-inflammatory response that involved the up-regulation of transcription levels of a wide range of chemokines, cytokines, their receptors, complement factors and other inflammation-related genes, the secondary response is characterized by an up-regulation of *ccr5*, *cxcr3*, *tlr13* and genes related to adaptive immunity, especially Ig chains. Within these Ig chains, both IgT and IgM seemed to play a similar role in terms of transcript levels and unique JSTs. Thus, although the trout gill response to other pathogens such as *Flavobacterium columnare* (22) or the parasite *Ichthyophthirius multifiliis* (12) revealed a preferential IgT response, in our study both Igs seem equally involved in the response to *L. petauri*. Although IgD is highly expressed in the gills of rainbow trout and Atlantic salmon, both of which contain an elevated number of IgD^+^IgM^-^ B cells (14, 16, 17), no regulation of IgD transcription was detected in response to *L. petauri*. In the case of both IgM and IgT, the BCR repertoire analysis performed indicated that the response was consistent with a clonal expansion of both types of Igs and, therefore, of both B cell lineages, but without evidence of clonal selection. The results presented demonstrated that local secondary responses are present in the gills upon re-infection, yet whether this is due to responses organized in remote tissues such as the spleen, or is a result of memory B and T cell populations retained in the mucosa should be further investigated. These studies are essential to understand how vaccines administered through mucosal routes in fish confer protection.

## Data Availability

The datasets presented in this study can be found in online repositories. The names of the repository/repositories and accession number(s) can be found below: (https://www.ncbi.nlm.nih.gov/geo/query/acc.cgi?&acc=GSE328875) under the GEO accession number GSE328875. The code for the bioproject is PRJNA1456916.
