# Supplementary material for: Transcriptomic profiling and IgM and IgT repertoire dynamics in rainbow trout gills following primary and secondary challenge with Lactococcus petauri
Source: Front Immunol. 2026 Jun 16;17:1863531. doi: 10.3389/fimmu.2026.1863531 (PMC13314482; doi:10.3389/fimmu.2026.1863531)
Supplement: Supplementary file 1 [file Presentation1.pptx]

## Slide 1
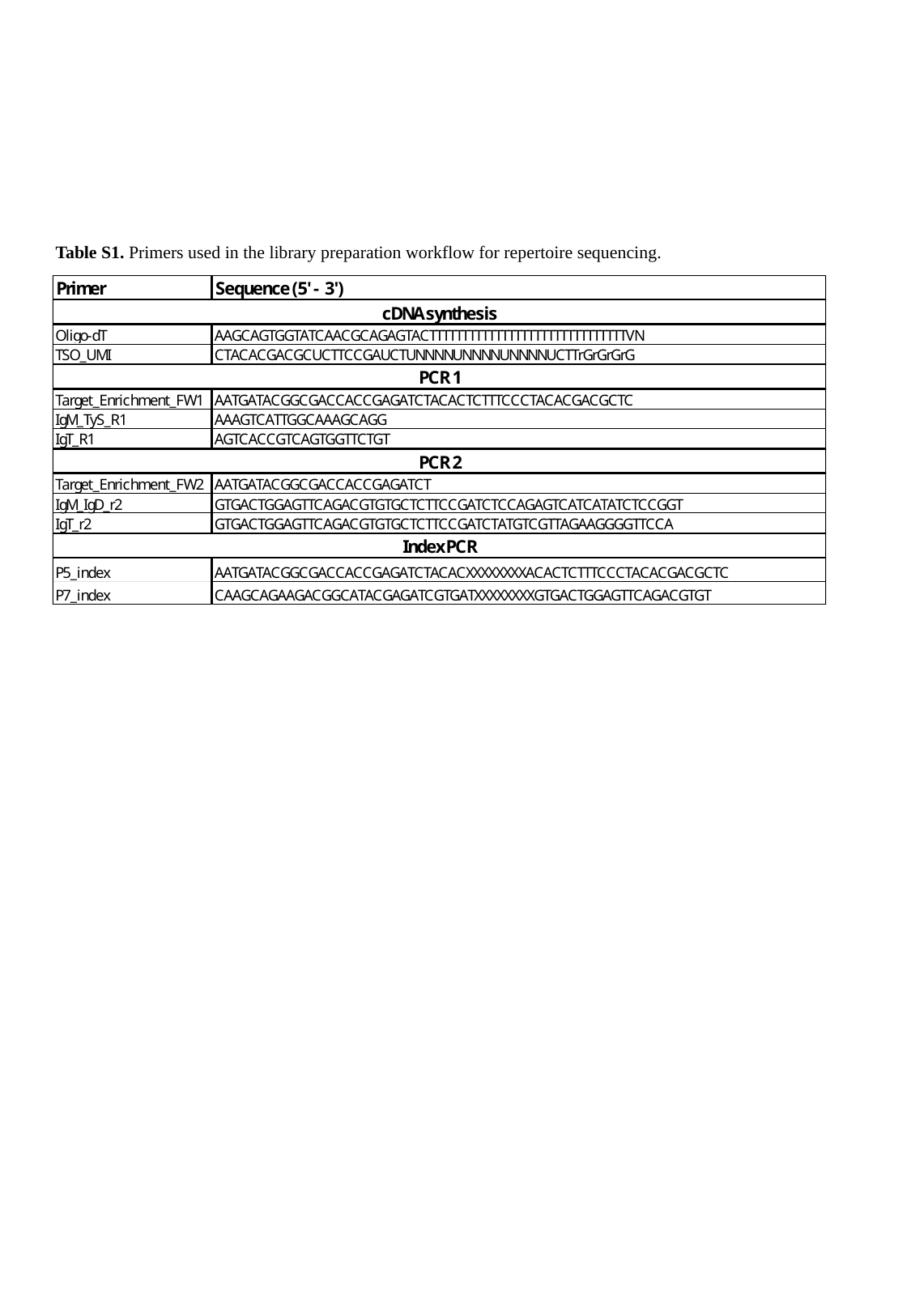

Table S1. Primers used in the library preparation workflow for repertoire sequencing.

## Slide 2
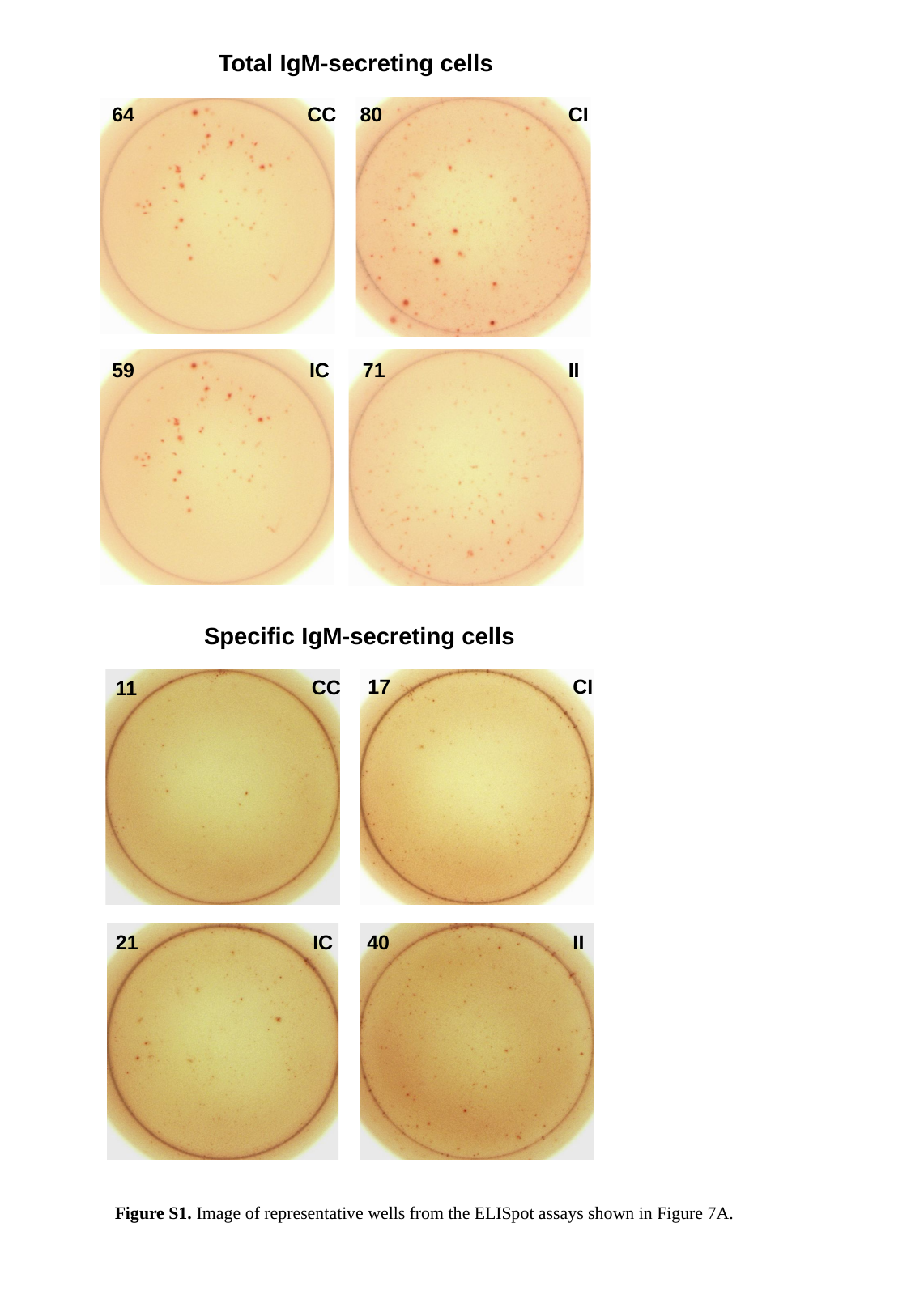

Total IgM-secreting cells
CC
80
CI
64
59
IC
71
II
Specific IgM-secreting cells
17
CI
CC
11
21
IC
II
40
Figure S1. Image of representative wells from the ELISpot assays shown in Figure 7A.
